# Structural and fluctuational difference between two ends of A*β* amyloid fibril: MD simulations predict only one end has open conformations

**DOI:** 10.1038/srep38422

**Published:** 2016-12-09

**Authors:** Hisashi Okumura, Satoru G. Itoh

**Affiliations:** 1 Institute for Molecular Science, Research Center for Computational Science, Okazaki, 444-8585, Japan; 2 The Graduate University for Advanced Studies (SOKENDAI), Department of Structural Molecular Science, Okazaki, 444-8585, Japan

## Abstract

A*β* amyloid fibrils, which are related to Alzheimer’s disease, have a cross-*β* structure consisting of two *β*-sheets: *β*1 and *β*2. The A*β* peptides are thought to be serially arranged in the same molecular conformation along the fibril axis. However, to understand the amyloid extension mechanism, we must understand the amyloid fibril structure and fluctuation at the fibril end, which has not been revealed to date. Here, we reveal these features by all-atom molecular dynamics (MD) simulations of A*β*42 and A*β*40 fibrils in explicit water. The structure and fluctuation were observed to differ between the two ends. At the even end, the A*β* peptide always took a closed form wherein *β*1 and *β*2 were closely spaced. The A*β* peptide fluctuated more at the odd end and took an open form wherein the two *β*-sheets were well separated. The differences are attributed to the stronger *β*-sheet formation by the *β*1 exposed at the even end than the *β*2 exposed at the odd end. Along with the small fluctuations at the even end, these results explain why the fibril extends from one end only, as observed in experiments. Our MD results agree well with recent observations by high-speed atomic force microscopy.

Amyloid fibrils, insoluble fibrous aggregates of misfolded proteins or peptides, are associated with approximately 40 human neurodegenerative diseases^1^,^2^,^3^,^4^. For example, Alzheimer’s disease is related to amyloid-*β* (A*β*) peptides, Huntington’s disease is caused by polyglutamine tracts, and dialysis-related amyloidosis is caused by *β*2-microglobulin. The A*β* peptide has 40–43 amino acid residues, and assembles into amyloid fibrils with a cross-*β* structure comprising two *β*-sheets, *β*1 and *β*2, as shown in Fig. 1(a) 5,6,7. The A*β* peptides arrange in an orderly array with the same confirmation along the amyloid fibril axis. Because the H and O atoms of the odd-numbered (even-numbered) residues in *β*1 are exposed at the right (left) side of this arrangement, this end is called the odd (even) end^8^. Although each end is known to expose a different side of the A*β* peptide to the solvent^6^, the different molecular structures and kinetics between the odd and even ends have not yet been reported.

An amyloid fibril extends by adding another peptide to its end^9^,^10^. To understand the molecular mechanism underlying amyloid fibril elongation, we must reveal the atomic structure of the fibril ends. This knowledge is particularly important for drug design as blocking the fibril ends would prevent fibril elongation. The fibril end forms the interface between the amyloid fibril and solution and may generally adopt a different molecular structure and character from the peptides in the bulk region. The scenario is similar to the surface reconstruction of crystals such as silicon or gold in solution^11^ and polarization on water surface^12^. In an amyloid fibril, the fibril ends interface with the solution, while the bulk comprises the center region of the fibril far from the ends. The atomic conformation in the bulk region has been experimentally elucidated by solid-state NMR spectroscopy. In contrast, the conformation at the fibril end has not been experimentally revealed because amyloid fibrils are essentially one-dimensional with zero-dimensional (vanishingly small) ends. Therefore, the fibril-end structure must be clarified by alternative techniques such as molecular dynamics (MD) simulation.

Several MD simulation studies have revealed the aggregation^13^,^14^,^15^,^16^,^17^,^18^,^19^,^20^,^21^,^22^,^23^,^24^ and disaggregation^25^,^26^,^27^ of A*β* amyloid fibrils, and a few MD studies have reported easy deformation of the A*β* molecules at the two fibril ends under high temperature (398 K) conditions^28^,^29^. However, the structural and fluctuational differences between the odd and even ends have not been investigated. To reveal these differences, the present study conducts MD simulations on A*β* amyloid fibrils composed of 20 A*β*42 peptides and 20 A*β*40 peptides in explicit water. We report, for the first time, the structural and fluctuational differences between the two ends of the A*β* amyloid fibril.

Surface science has become well established in solid-state physics and solution chemistry, but not in amyloid fibril research. In this study, we predict the A*β* molecular structures at the fibril ends, which have not been experimentally determined, from a surface-science perspective.

## Results and Discussion

### Structure and fluctuation of A*β* amyloid fibril

**Figure 1:**
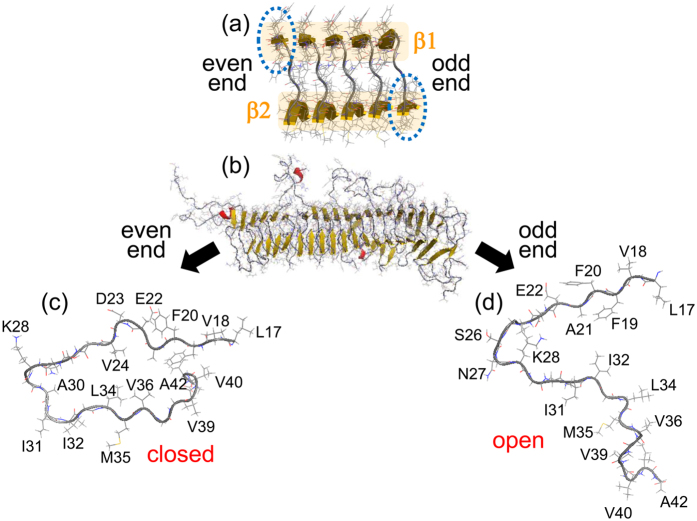
(**a**) Experimental conformation of the A*β*42 fibril (PDB: 2BEG). (**b**) Final conformation of one MD simulation. Side views of the A*β*42 monomer at (**c**) the even end and (**d**) the odd end. Residues D1 to K16 are not shown in the side views. The figures were created using PyMOL^60^. See Movie 1.

Movie 1 of Supplementary Information (SI) shows a typical MD simulation of the A*β*42 amyloid fibril. In the initial conformation, the two *β*-sheets were closely spaced. Some time later, the N- and C-termini opened at the odd end but remained closed at the even end (panels (b,c and d) of Fig. 1). In all simulations, the odd end often opened, whereas the even end never opened.

**Figure 2:**
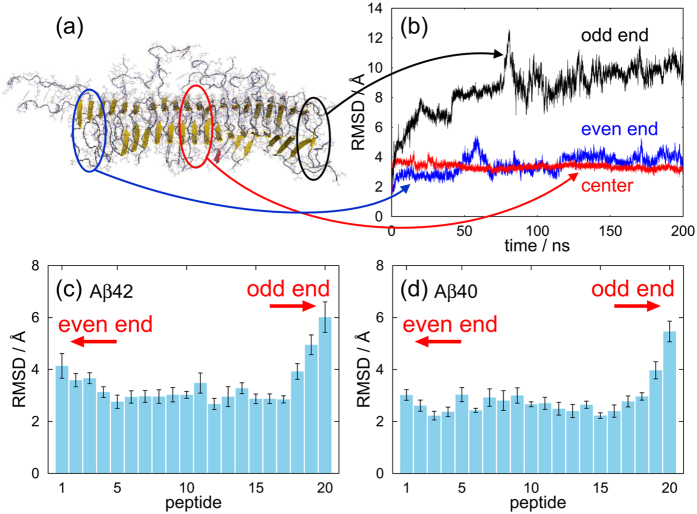
(**a**) Snapshot of a A*β*42 amyloid fibril. (**b**) Time series of RMSD of an A*β*42 peptide from the NMR conformation at both ends and in the center region, obtained from one MD trajectory. Average RMSD of (**c**) A*β*42 and (**d**) A*β*40 amyloid fibrils. (**a**) was created using PyMOL^60^.

Figure 2(b) shows time series of the root mean square deviations (RMSDs) of the A*β*42 peptides at both ends and in the center region. A typical snapshot is shown in Fig. 2(a). Fluctuations were largest at the odd end and slight at the center region and the even end. Figure 2(c and d) plot the average RMSDs over the nine initial conditions for A*β*42 and A*β*40, respectively, at times ranging from 100 to 200 ns. The fluctuations were clearly larger at the odd end than at the even end and were smallest in the center region. These differences were statistically significant and appeared in both A*β*42 and A*β*40 fibrils.

**Figure 3:**
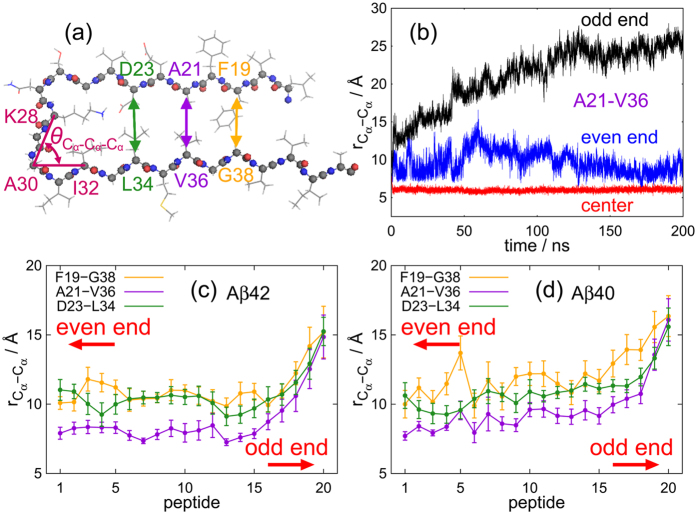
(**a**) Side view of the experimental conformation of the A*β*42 amyloid fibril (chain C of model 1 of PDB: 2BEG). (**b**) Time series of C_*α*_-C_*α*_ distance between A21 and V36 at both ends and in the center region. Average C_*α*_-C_*α*_ distances of the (**c**) A*β*42 and (**d**) A*β*40 amyloid fibrils between F19 and G38 (orange), A21 and V36 (purple), and D23 and L34 (green). (**a**) was created using PyMOL^60^.

Figure 3(b) shows the time series of the C_*α*_-C_*α*_ distance between A21 and V36 at both ends and in the center region, calculated during the MD trajectory of Fig. 2(b). The pair of C_*α*_ atoms of A21 and V36 is illustrated in Fig. 3(a). The C_*α*_-C_*α*_ distance notably increased with time at the odd end, fluctuated with no distinct temporal trend at the even end, and was essentially constant in the center region. Panels (c) and (d) of Fig. 3 plot the averages of three C_*α*_-C_*α*_ distances along the peptide lengths. At the even end, these distances were indistinguishable from those in the center region, indicating that the two *β*-sheets were closely spaced. At the odd end, these distances were noticeably greater, indicating that the *β*-sheets were well separated. Consistent with this finding, the N- and C-termini at the odd end were far apart, as shown in Fig. 1(d).

### Free-energy landscape

**Figure 4:**
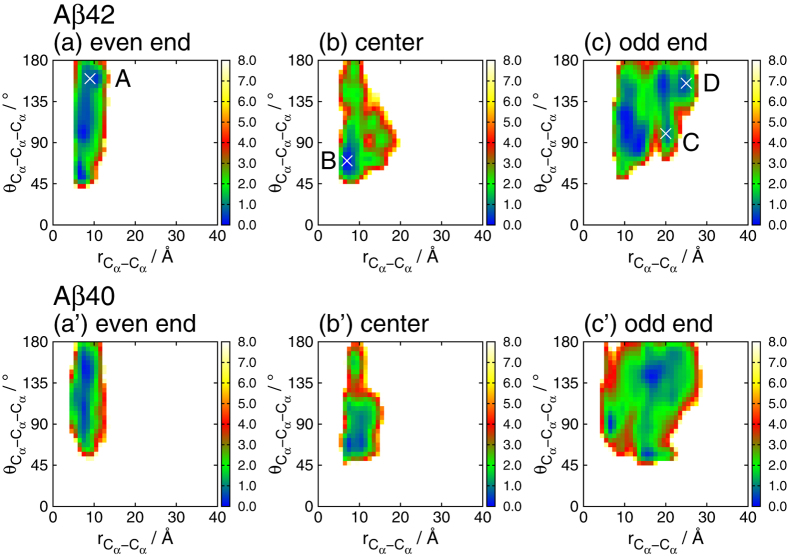
Two-dimensional free-energy landscape as a function of the distance 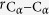 between the two C_*α*_ atoms of A21 and V36 and the angle 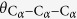 formed by the three C_*α*_ atoms of K28, A30, and I32 for the (**a**–**c**) A*β*42 and (a’–c’) A*β*40 amyloid fibrils (unit = kcal/mol) (**a** and a’) at the even end, (**b** and b’) in the center region, and (**c** and c’) at the odd end.

The two-dimensional free-energy landscape 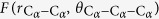 was calculated as a function of the C_*α*_-C_*α*_ distance 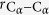 between A21 and V36 and the angle 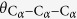 formed by three C_*α*_ atoms of K28, A30, and I32, as shown in Fig. 4. The angle 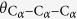, which indicates the swelling of the loop between the two *β*-sheets, was 68° in the NMR conformation (Fig. 3(a)). In the center region, the highly probable conformation was distributed around the experimental conformation of 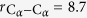 Å and 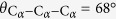, as shown in Fig. 4(b and b’). Although some probability distribution appears in the wide-angle area of 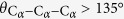, the probability is higher at both ends. The even end shows a similar vertically elongated landscape as the center region with high probability distributed throughout the wide-angle area of 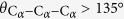. This landscape is broadly distributed over 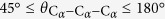. At the odd end, the free-energy landscape is widely distributed both vertically and horizontally. The C_*α*_-C_*α*_ distance between A21 and V36 can exceed 20 Å only at the odd end.

Because the odd end took both closed and open form, we calculated the the fractions of the open and closed forms, as listed in Table 1. Here, when the C_*α*_-C_*α*_ distance 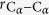 in Fig. 4 was longer than 20 Å, we regarded it as the open form. Free energy difference Δ*F*_closed→open_ from the closed forms to the open is also shown. Free energy difference Δ*F*_closed→open_ is the same order of room temperature (*k*_B_*T* = 0.59 kcal/mol), and the open form is often taken at the odd end.

**Figure 5:**
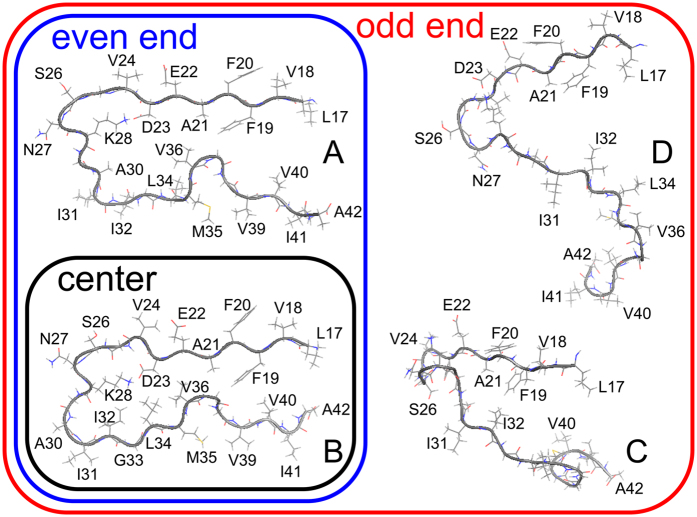
Typical conformations of the A*β*42 peptide. The figures were created using PyMOL^60^.

Figure 5 presents typical conformations at points A–D in Fig. 4(a–c). In conformation B, which is common in the bulk region, the N- and C-termini are closely spaced, and the side chains are closely packed between the two *β*-sheets, similar to the PDB conformation in Fig. 2(a). At the even end, conformations A and B are both observed. The loop region of conformation A is swollen, and some of its side chains (I32 and L34) protrude from between the two *β*-sheets. However, the N- and C-termini remain close, as in conformation B. The C_*α*_-C_*α*_ distance 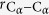 was almost identical at the even end and in the center region (Fig. 3(c)), despite the slightly larger RMSD at the even end than in the center (see Fig. 2(c)). This difference is attributed to fluctuations in the loop region. The even end fluctuates as conformation A while maintaining a short C_*α*_-C_*α*_ distance. Conformations C and D are found only at the odd end. The N- and C-termini are slightly opened in conformation C, and decidedly opened in conformation D.

### Reason why only the odd end opens

**Figure 6:**
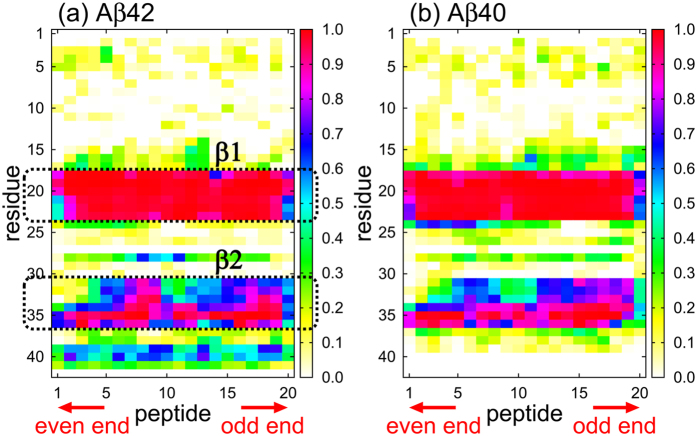
Probability that each amino acid residue in the (**a**) A*β*42 and (**b**) A*β*40 fibrils forms an intermolecular parallel *β*-sheet.

The larger fluctuations at both ends than in the bulk have been already reported in previous MD studies^28^,^29^. This finding is relatively trivial because both ends (unlike the bulk) are exposed to the solvent. However, the differences between the two ends are nontrivial. To understand these differences, we calculated the probability that each amino acid residue forms an intermolecular parallel *β*-sheet by using Define Secondary Structure of Proteins (DSSP)^30^. The probability distributions of the intermolecular parallel *β*-sheets are shown in Fig. 6. Because DSSP was used, formation of intermolecular *β*-sheet indicates that intermolecular hydrogen bonds were formed. In both A*β*42 and A*β*40 amyloid fibrils, residues V18 to D23 and I31 to V36 (delineated by the black dotted rectangles) form intermolecular parallel *β*-sheets with high probability. The former group is *β*1; the latter is *β*2. Because the glycine residue G33 in *β*2 takes a wide range of dihedral angles *ϕ* and *ψ*, the *β*1 region forms a *β*-sheet with higher probability than *β*2. According to the PDB conformation of Fig. 1(a), A*β* peptides are slightly twisted, and *β*2 does not lie directly below *β*1. Thus, *β*1 is more exposed than *β*2 at the even end, whereas *β*2 is more exposed than *β*1 at the odd end (indicated by the blue dotted ellipses in Fig. 1(a)). Both of these *β*-sheets, *β*1 at the even end and *β*2 at the odd end, might be easily broken in their environment. However, the amino acid residues in *β*1 form intermolecular hydrogen bonds with higher probability than those in *β*2. In general, a secondary structure with more hydrogen bonds fluctuates less than that with less hydrogen bonds^31^,^32^. In other words, the secondary structure with more hydrogen bonds is firmer than that with less hydrogen bonds. In this case, *β*1 fluctuates less than *β*2. *β*1 is like hard board, whereas *β*2 is like fluttering paper. *β*1 and *β*2 stick together by the hydrophobic interaction of their side chains^8^ except for *β*1 at the even end and *β*2 at the odd end because they are exposed to water. At the even end, *β*1 does not fluctuate much even if it does not sick with *β*2, because *β*1 forms firmer *β*-sheet by hydrogen bonds with the neighboring peptides. Figure 6 shows that *β*2 at the even end forms less intermolecular hydrogen bonds than *β*2 at the odd end. However, because it sticks with *β*1 of the neighboring A*β* peptide (i.e. 2nd peptide) by the hydrophobic interaction, *β*2 does not fluctuate much at the even end, either. On the other hand, because *β*2 at the odd end does not stick with *β*1 and forms less intermolecular hydrogen bonds than *β*1 at the even end, *β*2 at the odd end fluctuates more than *β*1 and *β*2 at the even end. Consequently, at the even end, where *β*1 is exposed, the A*β* peptide retains its closed forms (conformations A and B in Fig. 5) and constrains its fluctuations. At the odd end, where *β*2 is exposed, the A*β* peptide fluctuates comparatively widely and adopt many conformations, including the open conformations C and D.

We remark that the stagger^33^,^34^ of 2BEG, the model used here, is −1. That is, the N-terminus of A*β* peptide *i* interacts with the C-terminus of peptide *i* − 1 in the peptide numbering of Figs 2 and 3. There are other A*β* amyloid fibril models, such as 2LMN, 2LMO, 2LMP, etc., which have either more negative stagger or positive stagger. Depending on the sign of the stagger, *β*1 is exposed at the different end. Revealing which end opens in these models is a future research project.

Although the 3D coordinates of the A*β* peptides at the two fibril ends have not been experimentally determined, their different characteristics have been reported. Ban *et al*. observed that the A*β* fibrils extend only in one direction^35^,^36^. This unidirectionality of fibril extension implies different conformations of the odd and even ends, although the growing end has not been experimentally identified. Recently, Uchihashi and Konno observed a single amyloid fibril of yeast prion-protein sup35 by high-speed atomic force microscopy (AFM)^37^. Consistent with our MD simulations, they observed fluctuations at one end of the fibril; the other end remained steady. Furthermore, they observed that one sup35 molecule binds to the stable end, initiating elongation at that end. If the stable end also extends in the A*β* fibril, we can surmise that elongation proceeds from the even end, which fluctuated less than the odd end in our MD simulations.

In previous MD studies of A*β* fibril extension^38^,^39^, an A*β* peptide was added to either end of the fibril, while restraining the positions of the A*β* atoms at the end. According to Han and Schulten, the extension speed is 40 times faster at the even end than at the odd end because the additional A*β* peptide is locked in by the exposed hydrophobic residues at the even end^38^. However, Schwierz *et al*.^39^ reported a much smaller difference in the odd- and even-end extension speeds than that reported by Han and Schulten. In other simulations, residues already assembled into a *β*-strand easily form another strand with another peptide^19^,^21^,^22^. Our MD simulations revealed much less fluctuation of the even end than of the odd end, and stronger *β*-sheet formation by the *β*1 exposed at the even end. From these results, we infer that if an A*β* peptide is added to either end of the A*β* amyloid fibril without any restriction in the MD simulation, the extension speed would be much faster at the even end than at the odd end. We expect that the even end maintains a closed form in the existing intermolecular *β*-sheet and can readily form a new intermolecular *β*-sheet with the additional A*β* peptide. In contrast, the fluctuating odd end will less easily form a *β*-sheet with the additional A*β* peptide. The structural and fluctuational differences between the odd and even ends of the A*β* amyloid fibril may provide important insights into fibril extension.

## Conclusion

We revealed the structural and fluctuational differences between the even and odd ends of the A*β* fibrils in all-atom MD simulations. The even end always takes the closed form and fluctuates much less than the odd end. The fluctuating odd end can adopt both closed and open forms. The conformational flexibility of the odd end is attributed to the exposed *β*2, which forms weak hydrogen bonds. Meanwhile, the *β*1 exposed at the even end forms stronger hydrogen bonds and is more spatially constrained than the *β*2 exposed at the odd end. These results can explain the unidirectionality of fibril extension: The even end easily forms new *β*-sheets with another A*β* peptide, because it has already formed a stable *β*-sheet in the amyloid fibril. Our findings well agree with the results of recent high-speed AFM experiments.

By revealing the atomic structures at the fibril ends, we can design drugs that prevent A*β* amyloid fibril extension. Methods to inhibit the amyloid fibril formation have been attempted by many researchers^40^,^41^,^42^,^43^. From our MD simulations, the A*β* amyloid fibril is expected to elongate at the even end, where *β*1 is exposed. It may be a good strategy to design a molecule that binds to *β*1 for an inhibitor. The knowledge obtained from our simulations will be useful for understanding the amyloidogenesis mechanism and for overcoming amyloid diseases.

## Methods

### Modeling the initial conditions

The initial conformations of the amyloid fibril with 20 A*β*42 peptides, the amino-acid sequence of which was DAEFRHDSGYEVHHQKLVFFAEDVGSNKGAIIGLMVGGVVIA, were prepared as follows: model 1 of the 2BEG PDB file^8^ was used. Five A*β* (17–42) peptides formed intermolecular *β*-sheet structures between neighboring peptides in this PDB. The two edge peptides were removed because they were slightly distorted and unsuitable for a longer amyloid fibril. Ten copies of the other three peptides were aligned by the rigid translation so that intermolecular *β*-sheet structures between the trimers could be formed. By minimizing the potential energy with the conjugate gradient method in vacuum, an amyloid fibril with 30 A*β* (17–42) peptides was obtained. Ten of the 30 A*β* (17–42) peptides were then removed to obtain an amyloid fibril with 20 A*β* (17–42) peptides. Removing different ten peptides, three different conformations of 20 A*β* (17–42) peptides were obtained. The amino-acid residues 1–16, the conformations of which were not determined via the NMR experiments, were added with the dihedral angles of *ϕ* = *ψ* = 180°. However, these dihedral angles were not fixed at 180°, but flexibly fluctuated during the MD simulations. The residues 1–16 took random conformations and rarely formed secondary structures, as shown in Fig. 6. N- and C-termini of the peptide were left uncapped. The initial conformations of the amyloid fibril with 20 A*β*40 peptides were prepared in a similar manner. They were also obtained from model 1 of the 2BEG PDB file, but I41 and A42 were removed; the amino-acid sequence was DAEFRHDSGYEVHHQKLVFFAEDVGSNKGAIIGLMVGGVV. Employing three different initial velocities for each initial conformation, MD simulations were performed from nine different initial conditions for both A*β*42 and A*β*40 systems (18 initial conditions in total).

The A*β*42 amyloid fibril system consisted of 20 A*β*42 peptides, 57,876 water molecules, and 60 sodium counter ions. The A*β*40 amyloid fibril system consisted of 20 A*β*40 peptides, 58,015 water molecules, and 60 sodium ions. The total number of atoms was 186,228 and 186,065 for the A*β*42 and A*β*40 systems, respectively. A cubic simulation box was employed with periodic boundary conditions. The side length of the initial simulation box was *L* = 124.29 Å for both systems.

### Molecular dynamics simulations

MD simulations were performed by the Generalized-Ensemble Molecular Biophysics program developed by one of the authors (H.O.). This program has been applied to several biomolecules^44^,^45^,^46^,^47^. For the A*β* peptides and water models, we applied the AMBER parm99SB force field^48^ and the TIP3P rigid-body model^49^, respectively. The electrostatic potential was calculated using the particle-mesh Ewald (PME) method^50^. The cut-off distance was *r*_c_ = 12 Å for the Lennard-Jone potential. Temperature was controlled at 298 K using the Nosé-Hoover thermostat^51^,^52^,^53^, and pressure was controlled at 0.1 MPa using the Andersen barostat^54^. The symplectic^55^ quaternion scheme was used for the rigid-body water molecules^56^,^57^. Reversible multiple time-step MD techniques were also applied^58^. The time step was taken to be Δ*t* = 0.5 fs for the bonding interactions of the peptide atoms, Δ*t* = 2.0 fs for the non-bonding interactions of the peptide atoms and those between the peptide atoms and solvent molecules, and Δ*t* = 4.0 fs for the interaction between the solvent molecules. Because the symplectic rigid-body algorithm was used for the water molecules here, Δ*t* can be taken to be as long as 4.0 fs^57^. We performed an MD simulation for 200 ns from each initial condition. Averages of all physical quantities were taken over the last 100 ns and nine initial conditions.

### Analysis of the simulation results

Root mean square deviations (RMSD) in Fig. 2 was calculated for the backbone N, C_*α*_, and C atoms with respect to the reference conformation. Chain C of model 1 of the NMR structure (PDB ID: 2BEG) was used for the reference conformation^8^. Error bars of RMSDs and C_*α*_-C_*α*_ distances represent standard errors calculated using the bootstrap method^59^ for the nine MD simulations from different initial conditions. The number of bootstrap cycles was 1 × 10^7^.

Two-dimensional free-energy landscape 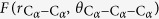 in Fig. 4 was calculated from the probability distribution 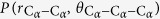 as a function of the C_*α*_-C_*α*_ distance 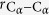 between A21 and V36 and angle 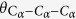 formed by three C_*α*_ atoms of K28, A30, and I32. It is given by


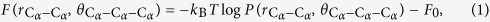


where *k*_B_ is the Boltzmann constant, *T* is a temperature of 298 K, and *F*_0_ is the minimum value of 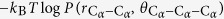. The landscapes at the even and odd ends were calculated from the probability distribution of one A*β* peptide at the respective ends. The landscape in the center region of the fibril was calculated by taking an average of six A*β* peptides in the center region.

Free energy difference Δ*F*_closed→open_ from the closed form to the open form in Table 1 was calculated from the fraction of these forms as


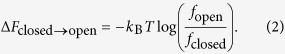


## Additional Information

**How to cite this article**: Okumura, H. and Itoh, S. G. Structural and fluctuational difference between two ends of A*β* amyloid fibril: MD simulations predict only one end has open conformations. *Sci. Rep.* **6**, 38422; doi: 10.1038/srep38422 (2016).

**Publisher's note:** Springer Nature remains neutral with regard to jurisdictional claims in published maps and institutional affiliations.

## Supplementary Material

Supplementary Information

Supplementary Movie 1

## Figures and Tables

**Table 1:** Fractions of the closed and open forms (*f*_closed_ and *f*_open_, respectively) at the odd end.

	*f*_closed_	*f*_open_	Δ*F*_closed→open_/(kcal/mol)
A*β*42	0.80 ± 0.11	0.20 ± 0.11	0.8 ± 0.3
A*β*40	0.79 ± 0.11	0.21 ± 0.11	0.8 ± 0.3

Free energy difference Δ*F*_closed→open_ from the closed form to the open form is also shown.
