# Supplementary material for: Structural and fluctuational difference between two ends of Aβ amyloid fibril: MD simulations predict only one end has open conformations
Source: Sci Rep. 2016 Dec 9;6:38422. doi: 10.1038/srep38422 (PMC5146922; doi:10.1038/srep38422)
Supplement: Supplementary Information [file srep38422-s1.pdf]

Supplementary information to "Structural and fluctuational difference between two ends of Aβ amyloid fibril: MD simulations predict only one end has open conformations"

Hisashi Okumura[1,2*] and Satoru G. Itoh[1,2]

[1]Institute for Molecular Science, Research Center for Computational Science, Okazaki, 444-8585, Japan
[2]The Graduate University for Advanced Studies (SOKENDAI), Department of Structural Molecular Science, Okazaki, 444-8585, Japan
*hokumura@ims.ac.jp

Supplementary Movie

Movie 1. Typical molecular dynamics simulation of the Aβ42 amyloid fibril.

Supplementary Figures

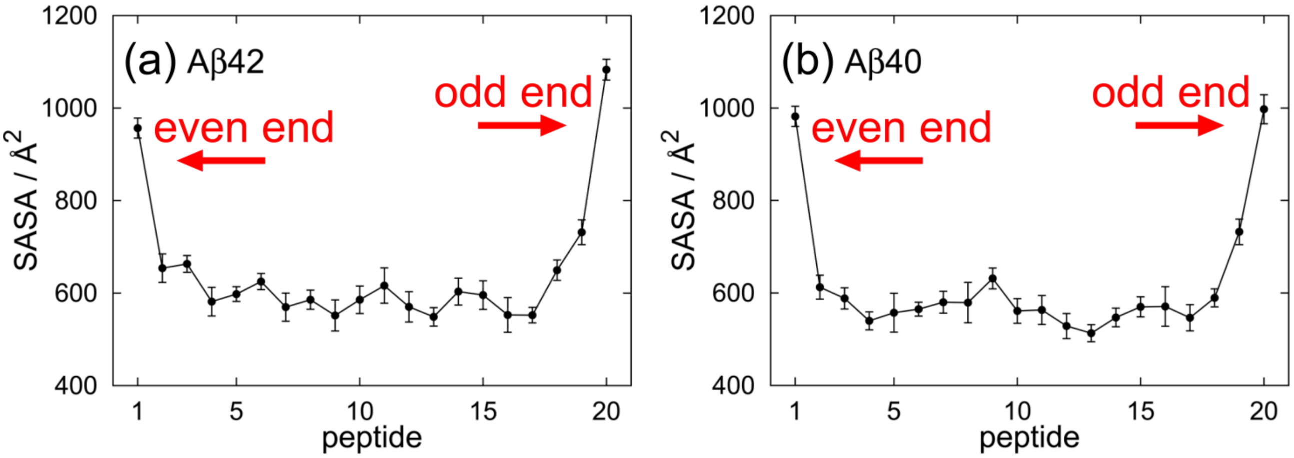

Figure S1. Solvent-accessible surface area (SASA) of each peptide in (a) the Aβ42 amyloid fibril and (b) the Aβ40 amyloid fibril.

Solvent-accessible surface area (SASA)[1] around the backbone atoms of each Aβ peptide was calculated, as shown in Fig. S1. SASA at both ends are larger than those in the center region. This is because both ends are exposed to water. Furthermore, SASA at the odd end is larger than that at the even end. This is clearly shown in the Aβ42 amyloid fibril, although it is less clear in the Aβ40 amyloid fibril. However, SASA of the second peptide from the odd end (i.e. 19th peptide) is larger than that of the second peptide from the even end (i.e. 2nd peptide) both in the Aβ42 and Aβ40 amyloid fibrils. This means that the odd end is open and water molecules can access into the inside of the amyloid fibril.

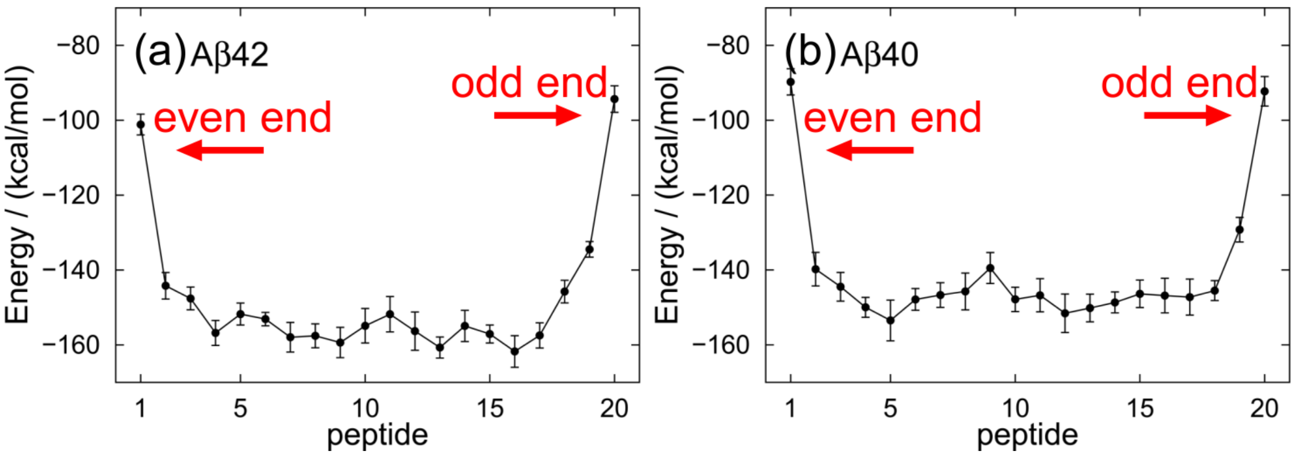

Figure S2. Lennard-Jones potential energy among peptide atoms in (a) the Aβ42 amyloid fibril and (b) the Aβ40 amyloid fibril.

Lennard-Jones potential energy among peptide atoms was also calculated, as shown in Fig. S2. Lennard-Jones potential energy E_i of peptide i is given by

$$E_i = \sum_{\alpha \in i} \sum_{\beta \neq \alpha} \frac{1}{2} 4\varepsilon_{\alpha\beta} \left\{ \left(\frac{\sigma_{\alpha\beta}}{r_{\alpha\beta}} \right)^{12} - \left(\frac{\sigma_{\alpha\beta}}{r_{\alpha\beta}} \right)^{6} \right\},$$

where α is an index for an atom in peptide i and β is that for an atom in any peptide. The constants of ε and σ are depth and diameter of Lennard-Jones potential energy, respectively. The distance between atom α and β is written as $r_{\alpha\beta}$. The difference of Lennard-Jones energy between the even end peptide (1st peptide) and odd peptide (20th peptide) is less clear because both peptides are exposed to water and the fluctuation of Lennard-Jones energy is larger than its difference between the open and closed forms. However, Lennard-Jones energy of the second peptides from the odd end (19th peptide) is higher than that of the second peptides from the even end (2nd peptide), as in the discussion on SASA.

References

1. Ooi, T., Oobatakake, M., Nemethy, G., & Scheraga, H. A. Accessible surface areas as a measure of the thermodynamic parameters of hydration of peptides. *Proc. Natl. Acad. Sci. USA* 84, 3086–3090 (1987).
